# Radiation Therapy for Primary Adenoid Cystic Carcinoma of the Trachea: Photons, Protons, or Carbon

**DOI:** 10.14338/IJPT-22-00036.1

**Published:** 2023-01-12

**Authors:** Alexander J. Tun, Bradford S. Hoppe, Yujie Zhao, Ian Makey, Sebastian Fernandez-Bussy, Xiaoying Liang

**Affiliations:** 1 Weinberg College of Arts and Sciences, Northwestern University, Evanston, IL, USA; 2 Department of Radiation Oncology, Mayo Clinic Florida, Jacksonville, FL, USA; 3 Division of Hematology Oncology, Mayo Clinic Florida, Jacksonville, FL, USA; 4 Department of Cardiothoracic Surgery, Mayo Clinic Florida, Jacksonville, FL, USA; 5 Department of Pulmonary Medicine, Mayo Clinic Florida, Jacksonville, FL, USA

**Keywords:** carbon, proton, trachea, adenoid cystic

## Abstract

Primary adenoid cystic carcinoma of the trachea (ACC-T) is an extremely rare cancer of the central bronchial system. It is usually associated with an excellent prognosis. Surgery is the standard treatment for resectable tumors, while radiation therapy is used for unresectable tumors or medically inoperable patients. Radiation therapy can be delivered with photons, protons, or carbon ion therapy. In this report, we review a case of unresectable ACC-T in a middle-aged female patient who was treated with radiation therapy and review the potential benefits of the different types of radiation therapy.

## Case

**Figure 1.**
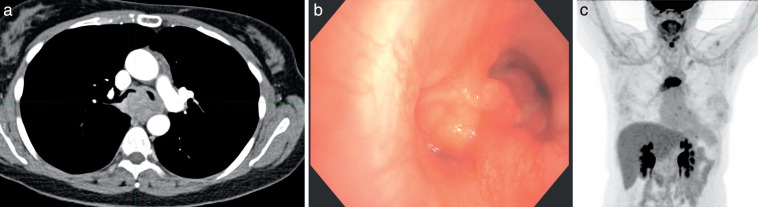
Initial evaluation of the adenoid cystic carcinoma on (a) contrast CT scan, (b) bronchoscopy, and (c) FDG-PET scan. Abbreviations: CT, computed tomography; FDG-PET, fluorodeoxyglucose positron emission tomography.

A 45-year-old woman was evaluated for progressive shortness of breath culminating in respiratory distress; a computed tomography (CT) scan showed a tracheal mass causing obstruction (**Figure 1a**). Bronchoscopy revealed an endobronchial tumor and extrinsic compression of 80% of the right mainstem bronchus, 90% of the bronchus intermedius, and 90% of the left mainstem take-off (**Figure 1b**). Tumor debulking with Argon Plasma Coagulation, electrocautery snare with balloon dilation, and stent placement in the right and left mainstem bronchi were performed. Pathologic features were consistent with ACC-T. A staging positron emission tomography–CT (PET/CT) scan revealed a hypermetabolic mass at the carina with extension into the right mainstem bronchus and bronchus intermedius with conglomerate hypermetabolic left lower paratracheal and subcarinal adenopathy (**Figure 1c**). While no standard staging has been developed for tracheal cancers, a modified TNM staging system would stage the patient's cancer as T2 (due to extension beyond the trachea into the carina) N1 (based on mediastinal lymph node involvement). The extent of involvement of the trachea made the tumor surgically unresectable; therefore, the patient was treated urgently with radiation and concurrent chemotherapy (weekly carboplatin AUC2 and paclitaxel 50 mg/m^2^ × 6 cycles). The patient was treated with a dose of 80 Gy to gross tumor volume and 70 Gy to the subclinical disease (clinical target volume) with a 5-mm uniform margin to the planned target volume, using a simultaneous integrated boost and volumetric modulated arc therapy (VMAT) over 40 fractions.

Primary malignant cancer of the trachea is extremely uncommon. An analysis of the National Cancer Institute SEER database spanning 3 decades (1973-2004) identified 578 cases of primary tracheal carcinoma at a median age of 63 years with 91% of patients older than 40 years. The most common was squamous cell carcinoma (44.8%), while adenoid cystic carcinoma of the trachea (ACC-T) was the second most common histologic subtype, accounting for 16.3% of the cases. Although the overall survival for the whole group is poor, with a 5-year overall survival of 27.1%, the survival associated with ACC-T is much higher at 74.3% [1].

The natural history of ACC-T is long and protracted as indicated by Maziak et al [2], who reviewed the cases of 38 patients with ACC-T treated at a large institution from 1963-1995. Thirty-two patients were treated with surgical resection and 6 patients were treated with radiation (50-75 Gy). The median overall survival was 60 months for the whole group, 93 months for the patients who had a complete resection, 61 months for patients with an incomplete resection, and 87 months for patients who received radiation therapy. Crude rates of local recurrence were 21%, including 9% after surgery and 83% after radiation therapy (50-75 Gy). Crude regional lymph node recurrence rate was 13%, while distant metastases rate was 45% of which 76% were pulmonary metastases.

**Figure 2.**
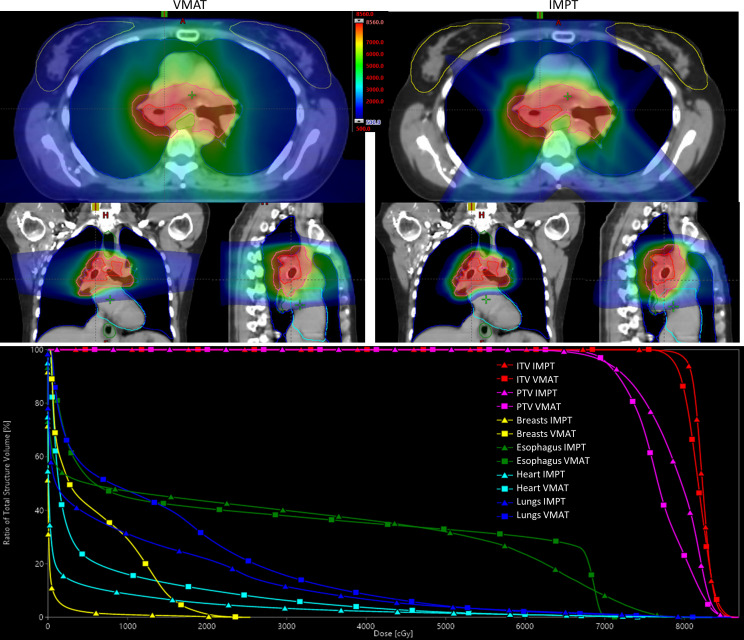
Radiation dose distribution for the VMAT plan on the left and the IMPT plan on the right, and dose-volume histogram on the bottom. Abbreviations: IMPT, intensity-modulated proton therapy; ITV, internal target volume; PTV, planning target volume; VMAT, volumetric modulated arc therapy.

Owing to the expected long survivorship of patients with ACC-T, we retrospectively replanned the patient by using intensity-modulated proton therapy (IMPT) to investigate potential benefits (**Figure 2**). Single-field optimization with 3 beams (anterior-posterior, right posterior oblique, and left posterior oblique) was used to generate the IMPT plan, so that each beam delivered a uniform dose to the treatment target. Proton therapy dose distribution is sensitive to the water-equivalent-thickness change along the beam paths owing to setup uncertainties; therefore, to achieve a robust plan against setup and range uncertainties, the IMPT plan was robustly optimized by using 5-mm setup uncertainty and 3.5% range uncertainty. Proton therapy did not substantially impact the mean dose to the esophagus (25.81 Gy IMPT versus 26.66 Gy VMAT) or heart (6.16 Gy VMAT versus 3.13 Gy IMPT). However, IMPT did significantly reduce the integral dose received by nontumor tissue (104.42 Gy × L VMAT versus 61.05 Gy × L IMPT), the mean lung dose (14.52 Gy VMAT versus 10.22 Gy IMPT), and the mean breast dose (5.89 Gy VMAT versus 0.48 Gy IMPT). This difference potentially could reduce the risk of radiation-induced breast cancer in this younger patient. In fact, one study reporting long-term outcomes for patients with ACC-T treated with radiation therapy included a secondary breast cancer within the photon radiation field [3]. Additionally, the lower dose to the lung could be important should oligometastatic treatment be needed to manage pulmonary metastases in the future either with resection or radiation therapy. Proton therapy has successfully been used and reported for a patient with unresectable ACC-T, with a combination of both photons and protons to a dose of 80 Gy with acceptable toxicities. The patient remained in complete remission at 11 months following the treatment [4].

Unfortunately, the patient in this case developed a local recurrence a year later, located within the high-dose region receiving 80 Gy. This is not surprising as ACC is known to exhibit radioresistance [5]. This led us to consider what alternative treatments we could have offered this patient, since surgery was not an option. Neutron therapy, a high linear energy transfer (LET) radiation modality, has been used in ACC of the salivary glands to try and overcome the radioresistance of this type of carcinoma [6]. However, late effects from neutron therapy have been quite high, which has diminished enthusiasm for the procedure, especially with only 1 center in clinical operation in the United States. On the other hand, carbon ion radiation therapy (CIRT), another type of high LET radiation, will be adopted by the Mayo Clinic in Jacksonville, Florida, and may provide the improved local control from neutron therapy but with the lower toxicities of proton radiation.

Heidelberg University has published its single institutional experience of treating ACC-T with CIRT in 7 patients [3]. Two patients received CIRT alone with a total dose of 60 to 63 Gy, while 5 other patients had a CIRT boost following photon therapy with a total dose of 68 to 74.4 Gy. After a median follow-up of 15.5 months there were no local recurrences or distant recurrences, but 1 patient (14%) did develop severe acute grade 4 stomatitis.

Our patient has been managed for her local recurrence with serial balloon dilations of the left mainstem bronchus and covered hybrid stent for the right mainstem bronchus. Additionally, she had oligometastatic progression with a solitary lung tumor in the left upper lobe and a separate lesion in segment VIII of the liver, which were both resected. She is currently being evaluated for a possible clinical trial.

In conclusion, we reviewed potential advantages of proton therapy and CIRT for a rare case involving a young female patient with unresectable ACC-T, including reduction in dose to normal organs with IMPT and CIRT and potential better local control with CIRT. Additional case series of ACC-T treated with particle therapy with longer follow-up are needed.
